# Translation, cultural adaptation and field-testing of the Thinking Healthy Program for Vietnam

**DOI:** 10.1186/1744-8603-10-37

**Published:** 2014-05-15

**Authors:** Jane Fisher, Hau Nguyen, Priya Mannava, Ha Tran, Thao Dam, Huong Tran, Thach Tran, Kelly Durrant, Atif Rahman, Stanley Luchters

**Affiliations:** 1 Jean Hailes Research Unit, School of Public Health & Preventive Medicine, Monash University, Melbourne, VIC, Australia; 2 Centre for International Health, Burnet Institute, Melbourne, VIC, Australia; 3 Research and Training Centre for Community Development, Hanoi, Vietnam; 4 Institute of Psychology, Health and Society, University of Liverpool, Liverpool, UK; 5 Department of Epidemiology and Preventive Medicine, Monash University, Melbourne, Australia; 6 School of Public Health, University of the Witwatersrand, Johannesburg, South Africa; 7 International Centre for Reproductive Health, Department of Obstetrics and Gynaecology, Ghent University, Ghent, Belgium

**Keywords:** Cultural adaptation, Field testing, Thinking Healthy Program, Maternal and child health, Vietnam

## Abstract

**Background:**

Depression and anxiety are prevalent among women in low- and lower-middle income countries who are pregnant or have recently given birth. There is promising evidence that culturally-adapted, evidence-informed, perinatal psycho-educational programs implemented in local communities are effective in reducing mental health problems. The Thinking Healthy Program (THP) has proved effective in Pakistan. The aims were to adapt the THP for rural Vietnam; establish the program’s comprehensibility, acceptability and salience for universal use, and investigate whether administration to small groups of women might be of equivalent effectiveness to administration in home visits to individual women.

**Methods:**

The THP Handbook and Calendar were made available in English by the program developers and translated into Vietnamese. Cultural adaptation and field-testing were undertaken using WHO guidance. Field-testing of the four sessions of THP Module One was undertaken in weekly sessions with a small group in a rural commune and evaluated using baseline, process and endline surveys.

**Results:**

The adapted Vietnamese version of the Thinking Healthy Program (THP-V) was found to be understandable, meaningful and relevant to pregnant women, and commune health centre and Women’s Union representatives in a rural district. It was delivered effectively by trained local facilitators. Role-play, brainstorming and small-group discussions to find shared solutions to common problems were appraised as helpful learning opportunities.

**Conclusions:**

The THP-V is safe and comprehensible, acceptable and salient to pregnant women without mental health problems in rural Vietnam. Delivery in facilitated small groups provided valued opportunities for role-play rehearsal and shared problem solving. Local observers found the content and approach highly relevant to local needs and endorsed the approach as a mental health promotion strategy with potential for integration into local universal maternal and child health services. These preliminary data indicate that the impact of the THP-V should be tested in its complete form in a large scale trial.

## Background

The common mental disorders of depression and anxiety are prevalent among women living in low and lower-middle income countries who are pregnant or who have recently given birth. A recent systematic review and meta-analysis concluded that on average in these countries, 16% of pregnant women and 20% of women caring for infants are experiencing clinically significant symptoms of a non-psychotic mental disorder, and that rates are much higher among the poorest women with the least access to health services and social protection [1].

Women with perinatal mental health problems (those occurring during pregnancy or up to one year postpartum) have reduced social and economic participation and are less able to adhere to recommended health care [2,3]. Poor maternal mental health is associated with lower sensitivity and responsiveness to infant behavioural cues and this compromised caregiving capability has flow-on consequences for the early growth and development of children. In resource-constrained settings maternal depression has been linked directly to higher rates of stunting, diarrhoeal diseases, infectious illness and hospital admission among infants, reduced completion of recommended schedules of immunization, and worse cognitive, motor, social, behavioural and emotional development in young children [4-9]. The adverse impacts of maternal common mental disorders on women and their children are exacerbated by poor socioeconomic conditions [10,11].

In Vietnam, which was recently reclassified by the World Bank from being a low to a lower-middle income country, perinatal mental health problems among women are highly prevalent. In a study using psychiatrist-administered individual diagnostic interviews to assess systematically recruited cohorts of women who were pregnant (n = 130) or had recently given birth (n = 234), 29.9% (95% CI = 25.2–34.7) were diagnosed with a common perinatal mental disorder. Prevalence was higher among women living in rural (Odds Ratio (OR): 2.2; 95% CI = 1.2–3.93) than urban areas [2]. These disorders have important consequences, with suicide accounting for up to 17% of pregnancy-related deaths in Vietnam in 2000 and 2001 [12]. Despite the high prevalence of perinatal mental disorders and their impact on maternal health, few services are currently available in Vietnam to address them. This is due in part to the competing health priorities of malnutrition and infectious diseases and the relatively low recognition of non-psychotic mental disorders, as well as the limited numbers of mental health professionals [11,13].

There is emerging evidence that this challenge can be overcome by using evidence-informed, perinatal psychological or psycho-educational programs in local communities, which have, if developed in other settings, been culturally adapted [14]. Rahman et al. [15] reviewed the thirteen available trials of these interventions that have been implemented in low- and middle-income countries. All had been delivered by trained, supervised health and community workers. Overall these had greater benefits for maternal mental health than routine care (pooled effect size for maternal depression was −0.38 (95% CI = −0.56 to −0.21). Some of the interventions also addressed caregiving skills and examined impact on child health and development. Where assessed, these included improvements in mother-infant relationship, better cognitive development and growth, reduced diarrhoeal episodes and increased immunization rates [15].

The highest quality study in the review was the cluster randomised controlled trial of the implementation of the Thinking Healthy Program (THP), by Lady Health Workers (LHWs) who are a cadre of community health workers, in rural Pakistan [16]. The THP was developed in a collaboration between the University of Manchester and the Human Development Research Foundation, Islamabad, Pakistan involving extensive community consultation including with mothers with and without depression, lady health workers, midwives, primary care doctors and traditional birth attendants [17]. The multimodal approach in the THP includes specific cognitive behavioural therapy (CBT) strategies to identify and modify maladaptive thinking styles, in particular fatalism and inability to act, superstitious explanations and somatization, to substitute more adaptive ways of thinking and to use behavioural activation to rehearse these strategies between sessions. It aims to improve women’s social status using the family’s shared commitment to the baby’s wellbeing as an entry point.

The THP program comprises five modules: preparing for the baby, the baby’s arrival and early, mid and late infancy. Each module contains sessions on the mother’s health, her relationship with her baby, and the relationships with people around her. The sixteen-session program is targeted at women with depression and their families, beginning around 30 weeks gestation and continuing to ten months postpartum. It was designed to be delivered in home visits by supervised lady health workers, who had received brief two-day training, strengthened by experiential learning and monthly half-day facilitated group supervision.

The impact of THP on perinatal depression was assessed in a cluster-randomized controlled trial in which Union Councils were allocated randomly to offer the intervention or enhanced standard care. Participants were married adult women, who met diagnostic criteria for depression and did not have serious physical morbidity or intellectual disability. At follow-up 12 months after implementation, it was found that compared to the control group, prevalence of depression among women in the intervention group was lower (27% versus 59%, Adjusted Odds Ratio (AOR) 0.23, 95% CI = 0.15-0.36); and they had less disability (Adjusted Mean Difference (AMD) -2.88, 95% CI = −3.66 to −2.10) and better global functioning (AMD 8.27, 95% CI 6.23-10.31). In addition, women who received the THP and their husbands spent more time playing with their children, whilst children had fewer episodes of diarrhoea and were more likely to have completed recommended immunization schedules [16]. These data indicate that it is feasible for community health workers to deliver THP in resource-constrained settings and that it is effective as a targeted individualised treatment for women who are experiencing perinatal depression.

The THP was developed for women living in Pakistan, where most people are Islamic and women’s lives reflect adherence to cultural and religious requirements including about clothing and appearance, participation in income-generating non-domestic work and autonomy to make decisions. It was designed as a treatment program for women diagnosed with depression. In order to be meaningful to women living in a different country and cultural context, and those with and without a mental health problem, adaptation is required. In order to increase reach it is also of interest to know whether it can be delivered effectively to groups of women and not just to individuals.

The overall aim of this study was to examine whether the THP could be applied in universal programs to improve population level perinatal mental health in Vietnam. The specific objectives were to investigate whether:

i. a translated and culturally adapted version of the THP was comprehensible, acceptable, and salient to women and community representatives in rural Vietnam;

ii. the THP-Vietnamese adaptation was safe and suitable for inclusion in a universal program to improve the psychological functioning of the whole population; and

iii. the THP-Vietnamese adaptation could be delivered effectively to small groups of women rather than to individuals.

## Methods

The project was undertaken (January to June 2013) in an international collaboration between the Research and Training Centre for Community Development (RTCCD) in Hanoi, Vietnam and the Jean Hailes Research Unit, Monash University School of Public Health and Preventive Medicine, and the Compass: Women’s and Children’s Health Knowledge Hub at the Burnet Institute in Melbourne, Australia with support from the Human Development Research Foundation in Islamabad, Pakistan and the Institute of Psychology, Health and Society at the University of Liverpool, England.

This study was undertaken in two stages:

Stage one: translation and adaptation

The first stage followed the World Health Organization’s Integrated Management of Childhood Illnesses (IMCI) Adaptation Guide steps 1 to 3.3 [18]: establishing an adaptation group; translation and checking for accuracy of language; identifying local terms for the conditions of interest; adaptation of materials to fit local idioms, images and circumstances, while maintaining essential components; consultation to reach consensus; and identification of needs to strengthen health or community worker capability. The process was informed by the World Health Organization’s guidance note *How IMAI (and IMCI) Support National Adaptation and Implementation of Task Shifting.*

Stage two: field test

Field-testing involves using and evaluating an intervention among a small group of potential end users in the setting it is ultimately designed for. Field testing is an early step in establishing the utility of an intervention for a new setting. It is intentionally subjective as it seeks to establish comprehensibility, acceptability, feasibility and salience. It cannot demonstrate impact and therefore does not require an adequately powered or representative sample. We field-tested the first four sessions of the translated and adapted THP (an introduction and the three sessions relating to mental health during the last trimester of pregnancy) in a rural commune.

### Setting

The field test was undertaken in Binh Nghia commune, Binh Luc district, Ha Nam province, which is a typical Red River delta rural area in Northern Vietnam. The population of Ha Nam is around 0.8 million people [19], and the average per capita income is USD 800 [20]. Most people live by subsistence farming, though some women are also employed in local industries and may undertake craft activities such as embroidery and basket weaving to generate income. Most pregnant women (99%) in Ha Nam register their pregnancies at a commune health station [2] and attend antenatal care at least once [19]. Vietnam has a highly structured national social organization, the Women’s Union, which has branches at all social levels, including local communes. It is ideally positioned and has strong capacity to engage women in activities related to women’s health and the family and represents a potential implementing workforce for the THP.

### Sampling and recruitment

Women aged at least 18 years who were registered with the Binh Nghia commune health station and at least 28 weeks pregnant were eligible to participate. All women meeting these eligibility criteria were informed of the study in home visits by the commune health worker and in announcements on the village loudspeaker system and invited to attend the commune health station for the sessions if they were interested to participate. Participants were given program materials to retain for personal use and were given a nutritious morning tea during the groups, and at the end of the program a small gift for their babies, but were not paid.

### Data sources

Brief self-report surveys which included both study-specific questions and, in some, a locally validated psychometric instrument.

#### Baseline survey

The baseline survey included structured, fixed-response option items assessing participant educational, occupational and marital status, household composition, pregnancy health and two screening questions for experiences of intimate partner violence. These questions were all drawn from surveys that we have developed and used in this setting before. We have established that these questions are understandable and meaningful to and can be answered by women living in rural areas in Vietnam [2,21]. It also included the 21-item version of the Depression and Anxiety Stress Scales (DASS-21-V) which has been formally validated against psychiatric interviews to establish sensitivity (79.1%), specificity (77.0%) and cut-off points (total score >33) to detect clinically significant symptoms of non-psychotic psychological morbidity among Vietnamese women in community settings [22].

#### Process surveys

Process surveys included study-specific, fixed choice questions to assess comprehensibility, salience, and acceptability of each session.

#### Endline survey

The endline survey included fixed-choice and open-ended questions to elicit appraisal of the whole series of sessions and suggestions for amendments. Psychological symptoms were re-assessed with the DASS-21-V.

### Procedure

All participants were given oral and written explanations of the study and asked to sign consent forms prior to study initiation. The baseline survey was administered at the beginning of the introductory session. The process surveys were administered after each session and the endline survey after the final session. Participants were encouraged to be frank in providing this feedback. Members of the Vietnamese adaptation sub-group attended and observed each session, took field notes and contributed to post-session discussions.

### Data analysis

The primary outcome of interest was feedback provided on the salience, comprehensibility, and acceptability of THP by the pregnant women and observers participating in the field test as well as from the discussion with mothers in Hanoi prior to the field test. Baseline and endline scores on the DASS-21-V were compared to identify indications of safety (no significant deterioration) and potential benefit (significant improvement).

### Ethics approval

Approval to conduct the study was obtained from the Ha Nam Provincial Department of Health Ethics Committee and the Women’s Union of Ha Nam.

## Results

### Stage one

The adaptation group included representatives of each partner agency, the Ha Nam Provincial Health Department, the Women’s Union, and women from the Vietnamese community who were pregnant or had recently given birth and were RTCCD staff members. It included specialists in mental health, women’s health and public health (some bi-lingual in Vietnamese and English) and Vietnamese clinicians, policy makers, potential implementers and end users.

The English language versions of the Thinking Healthy Program Handbook and Calendar (available at http://www.hdrfoundation.org) were made available to the adaptation group by the Human Development Research Foundation, Pakistan. Translation from English to Vietnamese and initial checking for accuracy of language and meaning were undertaken by bilingual health researchers. The Human Development Research Foundation assisted with clarification of intention where recommended strategies and activities specific to Pakistan had no direct Vietnamese equivalents.

**Figure 1:**
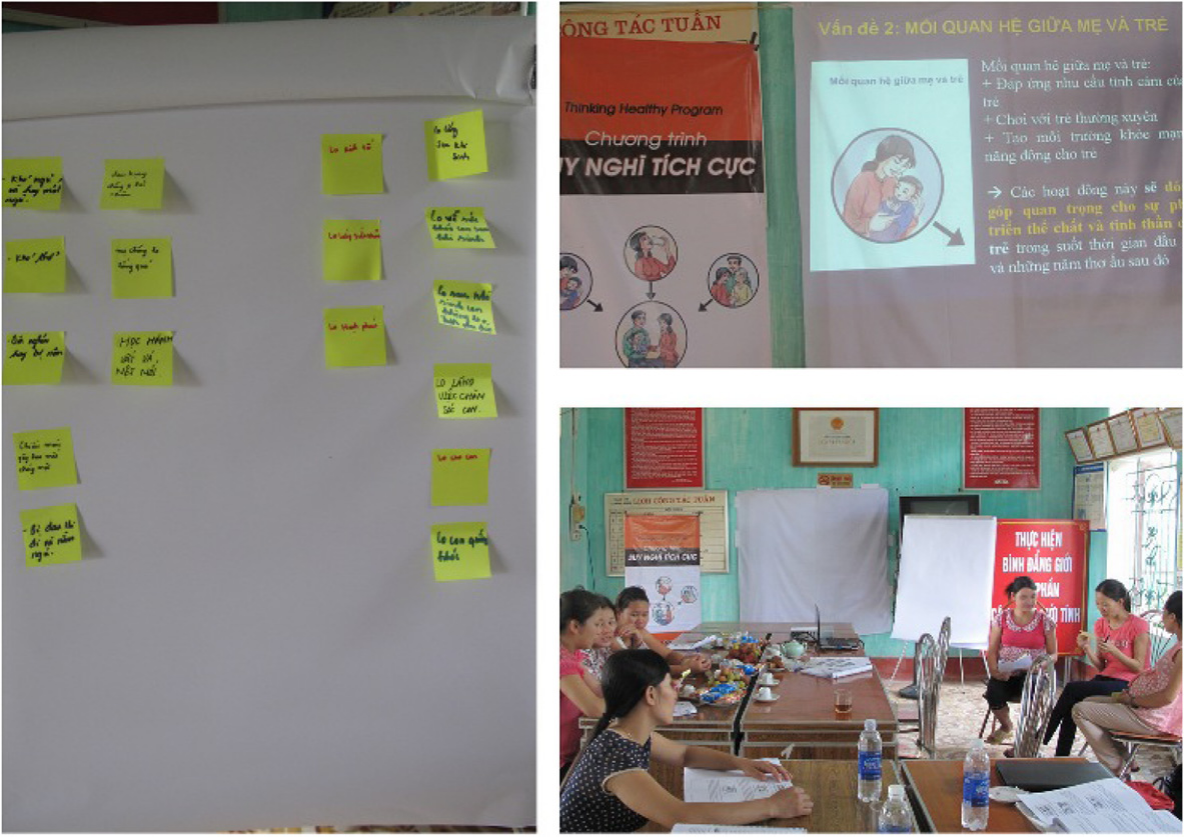
Training approaches used (clockwise from left): brainstorming (with flipchart), PowerPoint presentations and role-play.

Identification of local terms for the conditions of interest and further adaptation of materials to fit local idioms and imagery were undertaken by the Vietnam-based adaptation sub-group comprising health research workers qualified in public health and social sciences, many of whom also brought their perspectives as parents of young children. The local adaptation sub-group members were selected purposively for their knowledge of women’s health and social circumstances, maternal mental health, and their familiarity with the field site. The group identified cultural references that were not relevant to Vietnam and modified examples to fit local circumstances while maintaining essential components. The group then commissioned and supervised a Vietnamese artist to re-draw images to depict Vietnamese women, families and settings. The group reviewed the relevance and the applicability of the THP training structure and methodologies and identified health or community worker learning needs that would have to be addressed if the program were to be taken to scale. Finally the Vietnam adaptation sub-group developed versions of the materials that would enable them to be presented in a group rather than an individual format. These included use of sticky labels and a flip chart to record brainstorming ideas, a power point presentation and role-play activities (see Figure 1).

**Figure 2:**
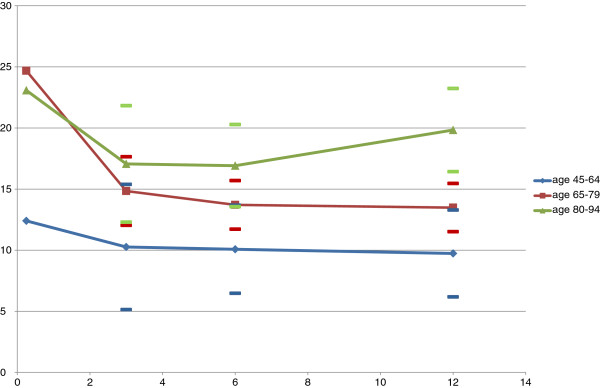
Image of woman from the Pakistan THP materials.

These processes identified the need to provide detailed guidance on the cognitive strategies that underpin the ‘thinking healthy’ approach because the notion of being able to change thoughts and behaviours was thought to be generally unfamiliar to community members and health workers in this setting. Participants also recommended review and revision of specific examples, pictures and materials provided in the THP to ensure that these were relevant to Vietnamese settings. These included changes to depiction of appearance (head covering is normative for women in Pakistan, but not in Vietnam) (see Figure 2), the use of brighter colours, and alteration of descriptions of cultural practices such as the “first month celebration” to fit local traditions and to remove suggestions that permission from a male family member was required in order to be able to leave the household. The mood-monitoring chart for completion between sessions at home was thought to be difficult and potentially unacceptable, as Vietnamese women do not necessarily reveal their personal experiences or emotions, particularly to strangers. It was proposed that time should be allocated to ‘experience sharing’ in which participants would be encouraged to share worries or concerns as an indirect way of gauging mood in relation to the particular perinatal period.

### Stage two

The field test involved delivery of the sessions to a small group of six pregnant women by three members of the Vietnamese adaptation sub-group, and observation by representatives of the international adaptation group, the commune health station and the Women’s Union. The facilitators were experienced in training using participatory techniques to optimise adult learning.

#### Participant characteristics

The characteristics of the group of pregnant women were assessed in the baseline survey and indicate that they were similar to the local population of pregnant women in terms of marital and occupational status, parity, and household composition (see Table 1). It appears that they were younger, more highly educated and less likely to be experiencing coincidental life adversity than the general population [2]. A score above the cut-off point of 33 on the DASS-21-V indicates clinically significant symptoms in this setting. The average score among the group was 14 (range 4 to 26) and no participant scored above 33.

**Table 1:** Baseline characteristics of six pregnant women who participated in the field test of the Thinking Healthy Program in Ha Nam province, Vietnam


Socio-demographic characteristics	
Mean age (range) years	21 (17 – 30)
Occupation (n)	
Farmer	3
Factory worker	2
Student	1
Education level (n)	
Completed secondary school	5
Vocational high school training	1
Marital status (n)	
Married	6
Perception of economic status (n)	
Similar to other households in this area	6
Parity (n)	
Nulliparous	4
Multiparous	2
Number of household members over prior six months, median (range)	5 (4 – 6)
Persons participant living with in the same household (n)	
Husband	6
Mother-in-law	6
Father-in-law	6
Husband’s brother/sister	2
Husband’s grandmother	1
Mother	2
Reproductive health	
Median gestation (range) weeks	32.5 (28 – 35)
Current pregnancy (n)	
Very welcome	6
Coincidental adverse events	
No current worrying or distressing coincidental life events	6
Relationship with partner	
Ever experienced fear of husband over the past year (n)	
Never	4
Sometimes or often	2
Ever physically hurt by husband over last year (n)	
Never	6

#### Process and endline evaluations of comprehensibility, acceptability and salience

All participants present at each session completed structured end-of-session evaluation surveys (see Table 2) and on completion of the whole of THP Module One, all six participants and the local observers completed program evaluation questions (see Table 3).

**Table 2:** Participants’ responses to fixed-choice questions on comprehensibility, acceptability, and salience of content of THP sessions 1 – 4

	**Session 1**	**Session 2**	**Session 3**	**Session 4**
	**(n = 6)**	**(n = 5)**	**(n = 6)**	**(n = 6)**
Phrase which best describes comprehensibility of session				
Easy to understand	1	1	1	1
Understandable	4	3	5	5
Somewhat understandable	1	1	-	-
Difficult to understand	-	-	-	-
Words which best describe content of session				
Very interesting		2	1	2
Interesting	5	3	5	4
Somewhat interesting	1	-	-	-
Not interesting		-	-	-
Program is relevant to*:				
Pregnant women	1	3	2	2
Women having young babies	1	3	2	2
Families of pregnant women or of women having infants (husband, parents/parents in laws)	5	4	5	5
Newlywed couples who plan to have a baby	-	-	-	-
Everyone older than 18 years in the village	-	2	-	-
Pictures convey key messages of the program				
Yes	6	5	6	6

*Could select more than one answer.

**Table 3:** Overall appraisal of Thinking Healthy Program Module One

	**Participants (n = 6)**	**Commune and Women’s Union representatives (n = 2)**
Value of activities done at home for putting into practice concepts covered in the session		
Extremely useful	5	2
Useful	1	-
A little useful	-	-
Not useful at all	-	-
The three step structure to thinking healthy made it easy to understand and practice healthy thinking and healthy actions		
Strongly agree	3	2
Agree	3	-
Neutral	-	-
Disagree	-	-
Strongly disagree	-	-
Comprehensibility of language used		
All understandable	6	2
Somewhat understandable	-	-
Somewhat difficult to understand	-	-
Mostly difficult to understand	-	-
Comprehensibility of content		
Easy to understand		-
Understandable	6	2
Somewhat understandable	-	-
Difficult to understand	-	-
Utility of program to pregnant women in the community		
Extremely useful	5	2
Useful	1	-
A little useful	-	-
Not useful at all	-	-
Most suitable trainers for the program		
Commune health station health worker	5	2
Village health worker	-	-
Commune women’s union member	2	1
Village women’s union member	-	-

### Comprehensibility

All participants found the sessions understandable, with an apparent increase in comprehensibility as sessions progressed, as none endorsed the ‘somewhat understandable’ category in the last two sessions. All agreed that the overall content of and language used in the program were understandable. One participant identified the main messages as having been how to (1) identify unhealthy thinking, (2) replace unhealthy thinking with healthy thinking, and (3) practicing healthy thinking and actions. Others provided shorter and more general answers, such as ‘positive thinking about mother’s relationship with her baby’ or ‘identifying negative thoughts about mother’s relationship with people around her’. The observers formed the view that participants understood the key messages of each session, and understood clearly the between session homework activities. The representatives from the commune health station and from the commune Women’s Union strongly agreed that the three-step approach facilitated understanding of the thinking healthy concept, and that the language and content of THP were understandable.

### Acceptability

Participants found the sessions interesting, with only one endorsement of the ‘somewhat interesting’ option throughout the program and it was for Session 1. Observers thought that all participants were engaged by the idea of responding actively to unhelpful or ‘unhealthy’ thoughts and realising that they could use active strategies to reframe thoughts to being ‘healthy’ ones. None of the women indicated that they disliked an aspect of any session.

### Salience

All the pregnant women reported that the skill of being able to identify unhelpful ‘unhealthy’ thoughts and replace them with healthy thoughts was relevant, useful and applicable to them, and that the three-step THP structure helped to enhance understanding of the concept. They agreed that the understanding and applying the cognitive and behavioural activation strategies was beneficial to their health as pregnant women. At least four women indicated that each session was relevant to women with young infants and their families. Some could see its wider relevance for other adults in the village. The Women’s Union and commune health station representatives indicated that the program would be extremely useful to pregnant women, and that all skills gained would be beneficial to maternal and child health.

### Suggested modifications to content and images

**Figure 3:**
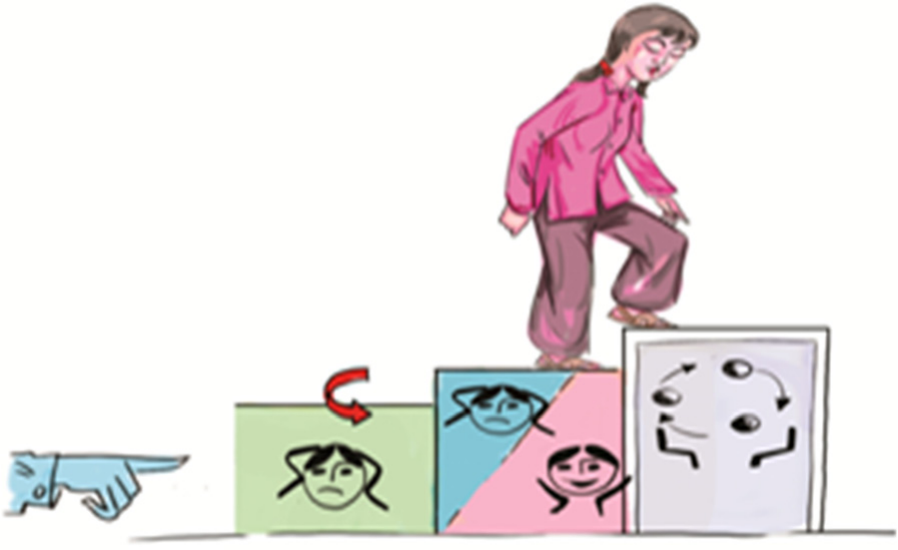
Image illustrating the three steps of the Thinking Healthy Program approach, but suggested to require modification for Vietnam to represent more optimistic body language.

In order to inform further development of relevant examples participants were asked to indicate the key factors influencing negative thinking in their daily lives. The following areas of concern were suggested to warrant explicit inclusion and consideration in THP-V. They also provided, in response to a request, suggestions for modifications (see Table 4 and Figure 3).

**Table 4:** Field-test participants’ suggested inclusions and modifications for the Thinking Healthy Program-Vietnamese adaptation (THP-V)


**Proposed inclusions**	
Session 1	Worries about personal health, relationships with family members, difficult socio-economic conditions, lack of confidence in personal abilities, and perceptions of a bleak future.
Session 2	Difficult socio-economic conditions, poor health, and relationships with family members.
Session 3	Difficult socio-economic conditions, unexpected pregnancy leaving the woman no choice but to accept her baby, and feelings of not wanting the baby or anticipated dislike of the baby.
Session 4	Difficult socio-economic conditions, looking after the baby increasing the mother’s work-load, pressures by people around the woman to diet, and lacking confidence or feeling inferior due to insufficient knowledge about pregnancy and taking care of a baby.
	All examples and pictures in Sessions 3 and 4 were endorsed as appropriate and relevant.
**Proposed modifications**	
Session 1	All participants stated strongly that the picture depicting a woman climbing the three steps to thinking healthy should be re-sketched to indicate optimism and hopefulness through positive body language.
Session 2	One participant felt that the example ‘my fate is to be sick’ as unhealthy thinking was not relevant and should be removed.

### Preferred facilitators for the THP

The pregnant women and the local representatives indicated that commune health workers or commune women’s union members would be the most suitable people to deliver the THP-V and that the structured content group facilitation skills could be conveyed in a short training program with follow-up supervision.

### Impact on mental health

THP group participants completed the DASS-21-V. There were no significant differences between mean baseline and mean endline (15.7, range 6 – 28) scores. No participants had scores in the clinical range at either assessment. A few had slightly higher endline than baseline scores; perhaps reflecting increased emotional literacy and self-awareness.

## Discussion

Overall this project has generated evidence that the translated and culturally adapted THP-V is acceptable and meaningful to pregnant women in a rural province in Vietnam. The language and content were found to be understandable and salient to participants, facilitators and observers, suggesting that the translated version of the original program materials from English to Vietnamese would be suitable for implementation. It was also found that it could be delivered effectively to small groups of women facilitated by a trained group leader and that role play, brainstorming discussions, and finding shared solutions to common problems were experienced by participants as valuable learning opportunities.

However, there is scope to enhance clarity of the content, in particular through the inclusion of examples suited to women’s predicaments in the Vietnamese context and more practical illustrative examples of how to use healthy thinking to address personal concerns. While the re-drawn images were found to be generally acceptable, there were some specific suggestions of how to improve them to engage interest and capture meaning more effectively.

The mean of the DASS-21-V scores did not change significantly between the baseline and endline assessments. There are several probable reasons for this. The first is that participants were not experiencing common mental disorders; their psychological functioning was in the non-clinical range and therefore there was limited potential for change in this domain. Second, the length of the field test (one month) was significantly shorter than that of the actual THP program (twelve months) and might not have been long enough to generate change in mental health. Third, only a quarter of the modules was delivered in the pilot, and therefore it might not have constituted an adequate ‘dose’. Fourth it is possible that participation in the program increased emotional literacy and raised awareness and this led to increased disclosure of distress among some by the end of the field test. Finally it is possible that as trust in the facilitators developed over the course of the study, participants were more able to be frank in their disclosures. Nevertheless we believe that the THP-V appears safe in this setting.

While the program was originally designed for the cultural and social context of Pakistan and as a treatment for depression, these preliminary data suggest that the underlying principles and the approach are relevant to all pregnant women and mothers of infants in resource-constrained settings including Vietnam. The program would be of value as a component of a package for universal implementation to improve the health of women during pregnancy and after giving birth, and the health and development of their infants. It was found that although confidence grew between sessions, it took time and effort to engage participants in these discussions initially. This could be addressed in facilitator training and in further adaptations of the program to ensure that there is a clearly staged structure.

Maternal health promotion in low-income settings has been confined to strategies to increase uptake of antenatal care, birth in medical facilities, improved nutrition and immunisation, but not to date mental health. In Vietnam almost all women attend for at least one antenatal visit and most give birth in medical facilities. Improving the mental health of women and children has recently been added to national policy priorities [23]. Vietnam has a strong primary health care system with commune level health centres staffed by trained health workers accessible to all. The Women’s Union is a highly structured national social organisation, with branches at all social levels, including local communes. Its mandate is to engage women in activities related to women’s health and the family. Together these indicate that primary health care and informal sectors are promising sites for the integration of mental health into maternal health promotion programs.

### Limitations

We acknowledge the limitations that because of resource constraints only the first four sessions of the THP-V, which pertain to pregnant women and not to women who have recently given birth, were tested. The sample was small and was not recruited systematically. It did not include women with current mental health problems or the complex personal predicaments of low literacy and very low household wealth and THP-V might not be suitable to their needs. In the small group format it was difficult to prevent women from talking to each other and comparing responses whilst filling out the session evaluation forms and the endline evaluation, so it is possible that they do not reflect individual opinions precisely. In order to learn about the facilitation and group processes, the THP groups were observed by up to two members of the adaptation group. We acknowledge that this might have influenced the behaviour of facilitators and participants, however, it was their impression that group discussions and activities were not constrained by their presence.

## Conclusion and implications for policy and practice

Overall these preliminary data indicate that the Vietnamese adaptation of the Thinking Healthy Program (THP-V) is safe for and comprehensible, acceptable and salient to pregnant women and also, it was suggested by participants, to women who have recently given birth and are caring for young children in Vietnam. Participants and facilitators valued the small group format and the opportunities it provided for role play rehearsal and shared problem solving. This suggests that the social support available in delivering THP to small groups rather than to individuals might enhance self efficacy compared to delivery in an individualised approach. Local observers who represented the commune health station and the Women’s Union also found the content and approach highly relevant to local needs and endorsed the approach as a mental health promotion strategy with potential for integration into local maternal and child health services.

## Abbreviations

AMD: Adjusted mean difference; AOR: Adjusted odds ratio; CBT: Cognitive behavioural therapy; CI: Confidence interval; DASS-21-V: The 21-item Vietnamese validation of the Depression Anxiety and Stress Scales; LHWs: Lady Health Workers; OR: Odds ratio; RTCCD: Research and Training Centre for Community Development; THP: The Thinking Healthy Program; THP-V: The Vietnamese adaptation of the Thinking Healthy Program; USD: United States Dollar.

## Competing interests

We declare that we have no competing interest.

## Authors’ contributions

JF, KD, and SL secured the grant; JF designed the research; HN and TDT translated the THP from English to Vietnamese; PM, HT, TD and HT contributed to the cultural adaptation, supervised the re-drawing of the images, trained and supervised the local facilitators, and conducted the field-test. AR developed the THP, made it available for this project and contributed to interpretation of the findings. JF and KD wrote the first draft of the paper. All authors were members of the adaptation group, contributed to manuscript revisions and approved the final manuscript.
